# Promoting Breastfeeding and Interaction of Pediatric Associations With Providers of Nutritional Products

**DOI:** 10.3389/fped.2020.562870

**Published:** 2020-11-25

**Authors:** Zsolt Bognar, Daniele De Luca, Magnus Domellöf, Adamos Hadjipanayis, Dieter Haffner, Mark Johnson, Sanja Kolacek, Berthold Koletzko, Miguel Saenz de Pipaon, Delane Shingadia, Pierre Tissieres, Luigi Titomanlio, Rezan Topaloglu, Johannes Trück

**Affiliations:** ^1^Pediatric Section of the European Society of Emergency Medicine (EUSEM), Paediatric Emergency Department, Heim Pal National Paediatric Institute, Budapest, Hungary; ^2^European Society of Paediatric and Neonatal Intensive Care, Paris Saclay University Hospitals-APHP, Medical Center A. Beclere, Clamart, France; ^3^Chair Nutrition Committee, European Society for Paediatric Gastroenterology, Hepatology and Nutrition (ESPGHAN), Department of Clinical Sciences, Umeå University, Umeå, Sweden; ^4^European Academy of Paediatrics (EAP), European University Cyprus, Paediatric Department, Larnaca General Hospital, Larnaca, Cyprus; ^5^European Society for Paediatric Nephrology (ESPN), Department of Paediatric Kidney, Liver and Metabolic Diseases, Children's Hospital, Hannover Medical School, Hanover, Germany; ^6^European Society for Paediatric Research (ESPR), National Institute for Health Research, Southampton Biomedical Research Centre, University Hospital Southampton NHS Foundation Trust and University of Southampton, Southampton, United Kingdom; ^7^European Society for Paediatric Gastroenterology, Hepatology and Nutrition (ESPGHAN), University Department of Paediatrics, Children's Hospital Zagreb, Zagreb, Croatia; ^8^European Academy of Paediatrics (EAP), Department of Paediatrics, University Hospital LMU Munich, Dr. von Hauner Children's Hospital, LMU-Ludwig-MaximiliansUniversität München, Munich, Germany; ^9^European Society for Paediatric Research (ESPR), Department of Pediatrics, Hospital Universitario La Paz, Universidad Autónoma de Madrid, Madrid, Spain; ^10^European Society for Paediatric Infectious Diseases (ESPID), Institute of Child Health, University College London, Great Ormond Street Hospital, London, United Kingdom; ^11^European Society of Paediatric and Neonatal Intensive Care, Paediatric and Neonatal Intensive Care Unit, Paris Saclay University Hospitals-APHP, Le Kremlin-Bicêtre, France; ^12^Pediatric Section of the European Society of Emergency Medicine (EUSEM), Pediatric Emergency Department and Migraine and Neurovascular Diseases Clinic, INSERM U1141—Paris University, Robert Debré Hospital, Paris, France; ^13^European Society for Paediatric Nephrology (ESPN), Department Pediatric Nephrology, Hacettepe University Faculty of Medicine, Ankara, Turkey; ^14^European Society for Paediatric Infectious Diseases (ESPID), University Children's Hospital Zurich, Zurich, Switzerland

**Keywords:** continuing medical education, infant nutrition, infant and young child feeding, public private sector cooperation, privately sponsored programs

## Abstract

Pediatric associations have been urged not to interact with and not to accept support from commercial providers of breast milk substitutes (BMSs), based on the assumption that such interaction would lead to diminished promotion and support of breastfeeding. The leadership of seven European pediatric learned societies reviewed the issue and share their position and policy conclusions here. We consider breastfeeding as the best way of infant feeding and strongly encourage its active promotion, protection, and support. We support the World Health Organization (WHO) Code of Marketing of BMSs. Infant formula and follow-on formula for older infants should not be advertised to families or the public, to avoid undermining breastfeeding. With consistently restricted marketing of BMSs, families need counseling on infant feeding choices by well-informed pediatricians. Current and trustworthy information is shared through congresses and other medical education directed and supervised by independent pediatric organizations or public bodies. Financial support from commercial organizations for congresses, educational, and scientific activities of pediatric organizations is an acceptable option if scientific, ethical, societal, and legal standards are followed; any influence of commercial organizations on the program is excluded, and transparency is ensured. Public–private research collaborations for improving and evaluating pharmaceuticals, vaccines, medical devices, dietetic products, and other products and services for children are actively encouraged, provided they are guided by the goal of enhancing child health and are performed following established high standards. We support increasing investment of public funding for research aiming at promoting child health, as well as for medical education.

During the last couple of years, different groups have tried to put increasing pressure on pediatric associations and demanded that these should not interact with commercial providers of nutritional products for infants and young children, and they should not accept any support from such companies for congresses, educational, or research activities. Some have proposed that “*pediatric associations should function without the influence of commercial interests*” (1). The underlying implication is that interaction with, and acceptance of, support by commercial enterprises, specifically dietetic companies, would change the actions of pediatric organizations such as educational activities and medical guidelines, and such interaction would lead to diminished promotion and support of breastfeeding. The leaderships of the European Academy of Pediatrics (EAP), the European Society for Pediatric Gastroenterology, Hepatology and Nutrition (ESPGHAN), the European Society for Pediatric Infectious Diseases (ESPID), the European Society of Pediatric and Neonatal Intensive Care (ESPNIC), the European Society for Pediatric Nephrology (ESPN), the European Society for Pediatric Research (ESPR), and the Pediatric Section of the European Society of Emergency Medicine (EUSEM) have individually and jointly reviewed and discussed these issues and came to a consensus on related concepts and policies. The aim of this position paper is to present our considerations and conclusions.

## Background

There is unanimous agreement that breastfeeding is the best way of infant feeding. It provides optimal protection and support for maternal and child health, including healthy child growth, development, and long-term health (2, 3). We strongly endorse proactive protection, promotion, and support of breastfeeding. Although breastfeeding rates and duration have increased considerably during the last few decades in many European countries, the current situation is not yet satisfactory, and considerable further improvements are possible and needed (4). Numerous factors may influence breastfeeding rates, for example, availability of information and support for parents and expectant parents, societal standards such as conditions of parental leave from work, support for breastfeeding in public and at the workplace, the practices of marketing breast milk substitutes (BMSs), and many others (4–8). Pediatricians and other health care professionals (HCPs) need to take responsibility and play an active role, particularly in informing, encouraging, and supporting parents and expecting parents.

In 1981 the World Health Assembly (WHA) adopted the *International Code of Marketing of Breast-milk Substitutes* (“Code of Marketing”) (9, 10). The global pediatric community is a strong supporter of this Code and its goal to eliminate improper practices of marketing of BMSs that may undermine breastfeeding. Since 1981, the WHA adopted several subsequent resolutions that refer to the Code of Marketing, including, in 2016, a resolution entitled “*Ending inappropriate promotion of foods for infants and young children*” (11). The text of this resolution states that the WHA” *WELCOMES with appreciation the technical guidance on ending the inappropriate promotion of foods for infants and young children*.” The chosen wording, “WELCOMES with appreciation” reflects the fact that some member states of the WHA raised reservations, and there was no full consensus on the resolution. WHA resolutions express opinions and may provide policy recommendations, but they are not binding for member states who retain their own responsibility for decision making on implementation of recommendations.

The World Health Organization (WHO) published the “*Guidance on Ending the Inappropriate Promotion of Foods for Infants and Young Children. Implementation Manual*” in 2017 (12). This guidance document refers to commercially produced foods marketed as suitable for feeding infants and children from 6 up to 36 months of age, including follow-on formula, the so-called “growing up milks” for toddlers, and commercial complementary foods. The guidance considers as BMSs “*any milks (or products that could be used to replace milk, such as fortified soy milk), in either liquid or powdered form, that are specifically marketed for feeding infants and young children up to the age of 3 years (including follow-up formula and growing-up milks)*.” It further states, “*Companies that market foods for infants and young children should not create conflicts of interest in health facilities or throughout health systems.”* Listed examples of actions considered inappropriate include, i.e., provision of free or reduced-price products considered BMSs through health workers or health facilities; donation of equipment, services, or gifts to health facilities, or HCPs; and sponsoring meetings of HCPs and scientific meetings. The background text proposes that the reputation and credibility of HCPs and their associations would be damaged if funding is accepted “*by a company that makes a profit on goods that are not in the best interest of maternal and child health*” (12), and it implies that breast milk substitutes generally would not serve the best interest of child health.

## Our Position

The conclusions presented here are based on extensive professional experience, a nonsystematic review of current literature, and detailed discussions within and among the participating societies, and they present a consensus position on concepts and policy.

We enthusiastically encourage effective protection, promotion, and support of breastfeeding. This requires active engagement of pediatricians and their collaborative action with multiple stakeholders in numerous areas such as policy and planning, parental leave, and other support of families, information, education and communication, training of HCPs and others, monitoring, and research (6). Improper marketing practices of BMSs can adversely affect breastfeeding practice, but the common assumption that marketing practices of BMSs would be the dominant factor predicting breastfeeding practices in Europe is not based on any accountable evidence. In fact, while the regulations and practices of BMS marketing are comparable in the European Union member states, breastfeeding rates and duration vary markedly between member states (4), which indicate that factors other than BMS marketing are of great relevance here. However, we do support a ban on advertising infant formula and follow-on formula for older infants to families and the public to avoid the risk of undermining breastfeeding. Infant formula and follow-on formula for older infants serve as substitutes for human milk, and improper marketing may mislead families to perceive formula feeding as equivalent to breastfeeding (6). While current European Union regulations restrict the marketing of infant formula, they do not do this to the same extent for follow-on formula. This is not satisfactory because follow-on formulas for older infants are generally presented with similar brand names and design as infant formulas. Therefore, advertising follow-on formulas can lead to cross-marketing of infant formulas. Also, no marketing to consumers should occur for foods for special medical purposes for infants (e.g., special formula for treating cows' milk allergy), which should only be used upon medical advice.

The WHO guidance document also suggested to ban all marketing for complementary foods and for formulas for young children (12). The WHO has defined complementary foods as all foods provided to infants other than human milk, which thus includes also infant formula (13), motivated by the goal to promote exclusive breastfeeding. In contrast, pediatric organizations and government bodies in Europe, such as the European Food Safety Authority, consider this definition unhelpful and even confusing. Given that many infants receive formula during the 1st year of life either alongside breastfeeding or as the sole diet, in Europe, complementary foods are generally defined as all solid and liquid foods provided to infants other than breastmilk, or infant and follow-on formula (14–17). From a pediatric perspective, homemade and commercial complementary foods are meant to be provided alongside breastfeeding and not as a replacement for breastfeeding, and hence, these are not considered as BMSs. Likewise, formulas for young children aged 1 year or older are meant to be offered as an alternative to cows' milk in a diversified diet to improve nutrient provision, along with continued breastfeeding, but not to replace breastfeeding. Therefore, complementary foods and formulas for young children do not require the same marketing restrictions as BMSs, provided there is no cross-promotion of formulas, with presentation under different product names and package designs. We see the risk that banning all information regarding complementary foods and formulas for young children may lead families to take uninformed inappropriate choices and could induce a preferential use of low-quality products without restricted advertising, such as soft drinks and junk foods. In the current situation with a very high prevalence of childhood obesity with its life-long adverse health consequences (18), this would be a highly undesirable effect.

Implementation of effective restrictions of the marketing of infant and follow-on formula to families and the public makes it essential that families can obtain evidence-based counseling on infant feeding choices from well-informed pediatricians and other HCPs. Therefore, pediatricians and other HCPs need access to evidence-based information on current scientific knowledge, as well as information on available products. Trusted information providers are learned pediatric societies and professional organizations, and governmental bodies. They share information with pediatricians and other HCPs through congresses, different forms of continuing medical education (CME), and other information channels. The essential role of pediatric professional organizations in providing guidance for the work of HCPs was again evident during the Covid-19 pandemic (19, 20). Medical professional societies are best placed to provide independent, unbiased, and effective CME.

We strongly disagree with the recently published position of the European pharmaceutical industry association taking the view that commercial enterprises should independently be organizing medical education activities (21). We maintain that commercial enterprises must not direct and offer CME activities for HCPs on their own because the existing conflicts of interest make it highly unlikely that bias can be avoided (22). Congresses and CME activities should therefore be organized under the direction and supervision, and within the regulatory framework of independent, nonprofit learned societies, professional organizations, or governmental and public bodies. The organizing bodies should follow core values such as humanity, integrity, quality, independence, respect, accountability, and transparency, ensure that programs and their contents are balanced and present evidence-based information, and strictly exclude any influence of commercial interests on the programs.

HCPs use commercial products and services in their work and require related information, including information on product properties from commercial providers, and they provide feedback to commercial manufacturers on medical needs. Therefore, a dialogue between HCPs and their organizations with commercial providers is helpful and necessary. A recently published survey reported that two thirds of pediatric associations in Europe accepted exhibition participation or other forms of support from commercial providers of BMSs (1) along with a variety of other sources of support, given that provision of quality information and CME requires considerable resources and funding. The collaboration between medical professional societies and commercial enterprises in congresses and education involves inevitable challenges, but also important opportunities (22). The mission, vision, and values of pediatric organizations should guide decisions on their interaction with commercial organizations, as expressed before by the American Academy of Pediatrics (23). The “*Code of Conduct for HCPs and Scientific Organizations*” adopted by the Biomedical Alliance in Europe and the “*Code for Interactions With Companies of the American Council of Medical Specialty Societies*” provide examples for possible guiding principles that should be followed (24). Pediatricians and other HCPs are aware that providers of pharmaceuticals and vaccines, medical devices, dietetic products, and other products and services, have commercial interests, and they are generally able to critically evaluate communication from commercial enterprises. We support the option of participation of commercial enterprises in commercial exhibitions held at medical meetings or their financial sponsorship for congresses, educational events, and other forms of continuing medical education (CME) for pediatricians and other HCPs under the direction of independent medical professional societies, or governmental and public bodies. However, event organizers need to follow agreed scientific, ethical, and societal standards (24), and ensure transparency and absence of any influence of commercial enterprises on the program and content of activities. We disagree with setting different rules and regulations for accepting support from companies offering dietetic products for infants and young children, compared to support from commercial enterprises offering other products, medications, and services for healthcare, considering that all commercial companies may have relevant conflicts with the interests of patients and the public that equally need to be carefully monitored and addressed.

Banning financial support from some commercial organizations, such as providers of BMSs, for activities of pediatric societies and professional associations, without an adequate replacement by public funding, would further disadvantage the provision of pediatric healthcare, compared to adult healthcare where such restrictions are not requested. Realistically, the expected financial losses cannot be compensated for by increased user fees or public funding. Therefore, it would lead to a much-reduced offer of, and access to, evidence-based information sharing to pediatricians and other HCPs, which they require on a regular basis to optimally support child health in their daily work. Moreover, the health policy of the European Union and European countries (25) and of the WHO (26) expressly encourage collaboration of all stakeholders in order to maximize impact and benefit for people.

The United Nations Convention on the Rights of the Child stipulates that children have the right to the highest attainable standards of health and health care (27). Accordingly, children deserve access to innovative, safe, and suitable pharmaceuticals, vaccines, medical devices, dietetic products, and other products and services that are appropriately evaluated. While we enthusiastically support breastfeeding, we recognize that healthy infants that are not (or not fully) breastfed need BMSs, and that many sick infants require therapeutic dietetic products, both of which should be of the highest achievable quality to support child health and well-being. We strongly disagree with the view expressed by the WHO that such products would generally be “*goods that are not in the best interest of child health*” (12). BMSs are needed for infants that are not breastfed for different reasons to support their health as best as possible (10). BMSs or supplements to breastfeeding can even be an essential medical requirement for some infants with certain disorders, such as those born very preterm and those with inherited metabolic disorders, intestinal failure, complex food allergy, congenital cardiac lesions, enterocolitic syndromes, severe gastroesophageal reflux, neurodisability, and others (28, 29).

European regulations stipulate the need for evaluating the safety and suitability of pharmaceuticals, medical devices, as well as dietetic products for infants. Such evaluations usually require clinical studies performed in public–private collaboration. We encourage and support public–private research collaborations for improving and evaluating health-related products and services for children, provided these are guided by prioritizing the goal of enhancing child health and are performed following current scientific, ethical, and societal standards (30). At the same time, we strongly recommend strengthening public funding investment for research aiming at promoting child health, as well as for CME, in the interest of all stakeholders.

## Conclusions

- Breastfeeding is the best way of infant feeding. We strongly encourage active promotion, protection, and support of breastfeeding, in particular through active encouragement and support by pediatricians and other HCPs.
- Breastfeeding rates and duration have increased during the last few decades in many European countries, but further improvements are needed and possible through collaborative efforts of pediatricians and multiple other stakeholders.
- Infant formula and follow-on formula for older infants may serve as breast milk substitutes and should not be advertised to families or the public, to avoid the risk of undermining breastfeeding.
- Complementary foods for older infants and toddlers as well as formulas for young children should be offered along with continued breastfeeding, but not replace breastfeeding. They do not require the same strict marketing restrictions as BMSs provided there is no cross-promotion of formulas for infants.
- Families need counseling on infant feeding choices by well-informed pediatricians and other HCPs who have access to current evidence.
- Pediatricians and other HCPs can obtain current information through congresses and other forms of continuing medical education that should be directed and supervised by independent pediatric organizations, or public bodies. Commercial enterprises should not direct and offer scientific events and CME for HCPs to avoid any bias due to potential conflicts of interest.
- Financial support from commercial enterprises for congresses, CME activities, and other activities of learned societies and professional organizations is an acceptable option if agreed scientific, ethical, societal, and legal standards are followed, which includes ensuring no influence of commercial enterprises on the program, and content of activities and transparency on financial support.
- Public–private research collaborations for improving and evaluating pharmaceuticals, vaccines, medical devices, dietetic products, and other products and services for children are encouraged, provided they are guided by the goal of enhancing child health and are performed following current ethical, scientific, and societal standards.
- Investment of public funding for research aiming at promoting child health, as well as for CME, should be strengthened.

## Author Contributions

ZB: study conception, design, and drafting the article. DD and MD: design and reviewing the article. AH: study conception and design, analyzing data, and drafting the article. DH, MJ, SK, MS, DS, PT, LT, RT, and JT: reviewing the article. BK: design, drafting, and reviewing the article. All authors read and approved the final manuscript.

## Conflict of Interest

The authors represent pediatric organizations that have all accepted support from a variety of commercial actors, including providers of nutritional products for infants and young children. MD received speaker fees from Abbvie AB, Arla Foods Ingredients, Baxter AB, Fresenius Kabi Sweden, Mead Johnson, Nestec Ltd. (Nestlé), Prolacta Bioscience, and Semper AB, one consultation fee from Danone Nutricia, and research grants to Umeå University from Baxter AB and Prolacta Bioscience. DD received research and educational grants from Chiesi Pharmaceuticals spa, ABBVIE Inc., Radiometer Ltd., served as consultant and lecturer for Airway Therapeutics, Chiesi Pharmaceuticals spa, ABBVIE Inc., Medtronic Inc., and MASIMO Inc., and has served on advisory boards for Chiesi Pharmaceuticals spa and ABBVIE Inc. He received travel grants and research technical assistance from Vyaire Inc., Osypka Medical los, and Sonoscanner Ltd. MJ has received conference travel reimbursements from Prolacta Bioscience. SK received lecture fees or research support assigned to the hospital from Abbott, AbbVie, Abela Pharma, Fresenius, Mead Johnson, Nestle, Nutricia, Oktal Pharma, and Shire. BK tends to be biased toward breastfeeding as member of the National Breastfeeding Committee, chair of the Nutrition Committee, German Paediatric Society, and President, Int Soc Research in Human Milk and Lactation. LMU—University of Munich and its employee BK received support for scientific and educational activities from the European Commission, H2020 Programmes DYNAHEALTH—633595 und Lifecycle—733206, the European Research Council Advanced Grant META-GROWTH ERC-2012-AdG—no. 322605, the Erasmus Plus Programmes Early Nutrition eAcademy Southeast Asia—573651-EPP-1-2016-1-DE-EPPKA2-CBHE-JP and Capacity Building to Improve Early Nutrition and Health in South Africa—598488-EPP-1-2018-1-DE-EPPKA2-CBHE-JP, the EU Interreg Programme Focus in CD-CE111 and the European Joint Programming Initiative Project NutriPROGRAM, the German Ministry of Education and Research, Berlin (Grant No. 01 GI 0825), the German Research Council (Ko912/12-1 and INST 409/224-1 FUGG), and from healthcare and nutrition companies, predominantly as part of publically funded research projects supported by the European Commission or German government. MS received lecture fees and travel reimbursements from Nutricia and Nestle. LT served as a scientific consultant for SHIRE Pharmaceuticals Group and the National French Haute Authorité de Santé. PT has received research grants from Chiesi Pharmaceuticals spa and bioMérieux Inc., and has been serving as consultant for Sedana Inc., Baxter Acute Therapies Inc., and Bristol Myers Squib Inc. JT received honoraria for advisory work from MSD Vaccines and travel reimbursements to scientific conferences from Takeda (formerly Shire) and GSK vaccines. None of the authors declares a conflict of interest as defined by the US National Academy of Medicine, with no circumstances involving the risk that the professional judgment or acts of primary interest may be unduly influenced by a secondary interest. The remaining authors declare that the research was conducted in the absence of any commercial or financial relationships that could be construed as a potential conflict of interest.

## Abbreviations

BMS: breastmilk substitutes
EAP: European Academy of Paediatric
EFSA: European Food Safety Authority
ESPGHAN: European Society for Paediatric Gastroenterology, Hepatology and Nutrition
ESPID: European Society for Paediatric Infectious Diseases
ESPNIC: European Society of Paediatric and Neonatal Intensive Care
ESPN: European Society for Paediatric Nephrology
ESPR: European Society for Paediatric Research
EUSEM: European Society of Emergency Medicine, Paediatric Section
HCPs: Health Care Professionals
MS: breastmilk substitutes
WHA: World Health Assembly
WHO: World Health Organization.
